# Art Perception in the Museum: How We Spend Time and Space in Art Exhibitions

**DOI:** 10.1177/2041669517694184

**Published:** 2017-02-01

**Authors:** Claus-Christian Carbon

**Affiliations:** Department of General Psychology and Methodology, University of Bamberg, Bavaria, Germany; Forschungsgruppe EPÆG (Ergonomics, Psychological Æsthetics, Gestalt), Bamberg, Germany; Bamberg Graduate School of Affective and Cognitive Sciences, Bavaria, Germany

**Keywords:** empirical aesthetics, museum context, ecological perception, artworks, aesthetic appreciation, time, Gerhard Richter, insights, incubation, viewing distance, canvas size, area size, art viewing distance accommodation

## Abstract

Aesthetics research aiming at understanding art experience is an emerging field; however, most research is conducted in labs without access to real artworks, without the social context of a museum and without the presence of other persons. The present article replicates and complements key findings of art perception in museum contexts. When observing museum visitors (*N* = 225; 126 female, *M*(age) = 43.3 years) while perceiving a series of six Gerhard Richter paintings of various sizes (0.26–3.20 sq. m) in a temporary art exhibition in January and February 2015 showing 28 paintings in total, we revealed patterns compatible to previous research. The mean time taken in viewing artworks was much longer than was mostly realized in lab contexts, here 32.9 s (*Mdn* = 25.4 s). We were also able to replicate visitors spending more time on viewing artworks when attending in groups of people. Additionally, we uncovered a close positive relationship (*r*^2 ^= .929) between canvas size and viewing distance, ranging on average between 1.49 and 2.12 m (*M* = 1.72 m). We also found that more than half of the visitors returned to paintings, especially those people who had not previously paid too much attention at the initial viewing. After adding the times of returning viewers, each picture was viewed longer than had been estimated in previous research (*M* = 50.5 s, *Mdn* = 43.0 s). Results are discussed in the context of current art perception theories, focusing on the need for the ecologically valid testing of artworks in aesthetics research.

In 2001, Smith and Smith presented a seminal article on empirical aesthetics to provide data on viewing times in a museum context according to some key variables such as age, gender, and group size. The article has been widely referenced ever since, as it was the first systematic research to document a much longer elaboration of artworks in museums: On average 27.2 s longer, in fact, than in typical lab settings where usually less than 3 s are realized. Subsequent critical voices have repeatedly argued that the very different viewing times and viewing conditions in museums make it difficult to assess the ecological validity of such lab studies in which artworks are presented on computer monitors, and as such they lack authenticity (Wolz & Carbon, 2014), merely showing depictions of artworks at very different, mostly standardized sizes (Locher, Smith, & Smith, 2001). Research has repeatedly documented the effects of viewing and therefore also usable processing time on the specific perception, elaboration, and understanding and appreciation of art. In their seminal microgenetic approach on art perception, Bachmann and Vipper (1983) showed a clear influence of presentation time on the overall perception of art; a finding that was later specifically tested by Augustin, Leder, Hutzler, and Carbon (2008) with regard to the dimensions of content and style of art. It was revealed that information on style was only available after processing of content issues had been started (Augustin et al., 2008), a finding which was further demonstrated via an Event-Related Potentials (ERP) study making use of lateralized readiness potentials combined with the N200 effect (Augustin, Defranceschi, Fuchs, Carbon, & Hutzler, 2011). Even the differential viewing time conditions of artwork titles had a clear impact on the understanding of artworks—people with a deeper knowledge of art especially benefitted from longer viewing times (10 s compared with 1 s) of the titles (Leder, Carbon, & Ripsas, 2006). This result is of particular interest here, as the 10-s viewing condition is much closer to natural viewing conditions in museums than those typically realized in lab-oriented research (Smith & Smith, 2001).

It is certainly very important to consider the impact of viewing *time* on art perception, but this is just a part of the whole story of art experience. Another facet is represented by viewing *distance*. The viewing distance is usually fixed in experimental research, especially in lab research presenting depictions of artworks on computer monitors. When entering a museum, however, it soon becomes quite obvious that visitors change their viewing distance from art piece to art piece in a rather intuitive way. Systematic information on typical, self-chosen distances which visitors use to inspect an artwork is mainly missing. Such data are highly important to adequately simulate typical museum behaviour in the lab; such data should also provide some information on how perception potentially changes with the size of the observed painting and thus how we might adjust distances in the lab to be in accord with such practices (cf. Locher et al., 2001).

Last but not least, Smith and Smith (2001) have already drawn our attention to another important property of museum visitors: In contrast to participants in typical lab studies being tested individually to guarantee an unbiased, personal evaluation, visitors to museums often gather together in social groups in front of artworks: Smith and Smith documented that about ¼ of all study attendees visiting the exhibition were with one other person (19.3%) or with two other people (3.3%). The interaction between visitors while viewing an artwork might change the art experience as well, generating a social event out of it.

The present study aimed to replicate and expand on the study of Smith and Smith (2001) for several reasons: (a) since its publication in 2001, the Smith and Smith study has been a kind of gold standard of reference when discussing and referring to typical viewing times in ecologically valid art contexts, so any kind of replication seems to be mandatory; (b) the Smith and Smith study was conducted in the permanent collection of the Metropolitan Museum of Art (the MET) in New York city, one of the largest art museums in the world, so viewing times could have been biased by the specific (probably touristic) behaviour of visitors trying to catch a very long list of masterpieces within a narrow global time frame; (c) the Smith and Smith study lacked information on viewing distance and how often artworks were reassessed by multiple viewings of the artworks. Accordingly, we conducted an observation study on an art exhibition with a very limited number of paintings; in actuality this was a temporary exhibition in one big exhibition hall solely devoted to one painter (Gerhard Richter), to test explicitly for a very different character of exhibition and to gain more control of (multiple) visitor viewings of the artworks. To fulfil these challenging observation duties, we employed a state-of-the-art observation tool which we programmed as an Android app where viewing time, viewing distance, and further variables—aside from demographic data—were traced; among them how often the registered visitors returned to some paintings.

## Study

In this observation study, we tested some of the findings revealed and the hypotheses put forward by the seminal article of Smith and Smith (2001). The main aim was to analyse museum visitors’ behaviour in terms of viewing duration and distance, how often people returned to a painting and how behaviour changed throughout such reassessments.

## Method

### Participants

We tested a total of 225 visitors (126 female, *M*(age) = 43.3 years) attending the special exhibition on Gerhard Richter by unobtrusively observing them from a balcony above, which was barely detectable by typical visitors; 104 people visited the paintings on their own (category *single*), 100 visited them with one other person (category *pair*), 11 in a group (category *group*) of two or more, and 10 with their children (category *family*—here the children were not observed further, but a focus was set to the person who first attended the respective artwork). A total of four persons attended the exhibition with a wheelchair, two with a folding chair, and one with a walking stick; no other accessories in this respect were recorded. None of the participants detected the observers and so were naïve to the purpose of the study.

### Stimuli and Apparatus

Six paintings (see Table 1) by Gerhard Richter, a German visual artist born in 1932 in Dresden or German Empire, known worldwide for his abstract—as well as photorealistic—artworks, which realized the highest auction prices ever for a living artist. All were displayed at a temporary exhibition called *Detail: Paintings from the Böckmann Collection* at the Neues Museum Nürnberg (NMN; State Museum for Art and Design in Nuremberg) in Nuremberg, Germany. The whole exhibition was defined as a comprehensive and representative selection of Richter’s work. It comprised 28 works in total.

**Table 1.** List of Artworks for Which the Viewing Behaviour Was Registered.

No.	Title	Year	Style	Width (cm)	Height (cm)	*M* (complex)	*M* (liking)	*M* (ambiguous)	*M* (interesting)
1	Abstraktes Bild	1989	Abstract	125	100	4.5	4.6	4.6	4.8
2	Krems	1986	Abstract	72	102	5.2	4.4	4.7	5.1
3	Abstraktes Bild	1988	Abstract	200	160	4.3	2.7	3.6	3.2
4	Decke	1988	Abstract	200	140	3.8	3.2	3.9	3.4
5	Abstraktes Bild	1999	Abstract	55	48	4.8	3.1	4.5	3.6
6	Blumen	1994	Figurative or photorealistic	51	71	3.1	3.2	3.3	3.3

*Note.* All paintings were by Gerhard Richter, painted in oil on canvas, with the exception of no. 5 being painted in oil on Alu Dibond. The four variables *complex*, *liking*, *ambiguous*, and *interesting* originated from a post hoc study which was conducted with a different sample of *N* = 10 (six female, *M*(age) = 24.2 years) who were presented the paintings in blocks in the order indicated below on Likert ratings from 1 (*not very much*) to 7 (*very much*).

**Figure 1.**
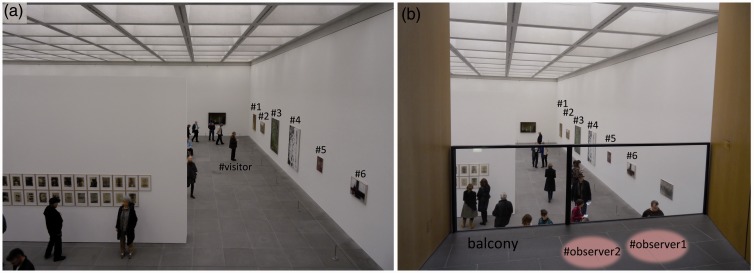
Original setting in the exhibition hall. (a) The order of focused paintings in detail, seen by the observers located at the balcony upstairs with a typical visitor standing in front of painting no. 3 at a viewing distance of 1.5 m (equals three tiles away from the painting’s plane) and (b) the same setting including the approximate locations of the two observers on the balcony, indicated by two ellipses titled Observer 1 and Observer 2.

The six paintings which were utilized for the study were all positioned side by side on one wall of the only hall in the entire exhibition; the two observers assessing visitor behaviour were situated on a balcony above the hall overseeing the entire scene of interest (for an original setting please refer to Figure 1). On the floor, the tile sizes were exactly 50 × 50 cm, allowing the easy assessment of viewing distance between visitor and painting with a resolution of 50 cm accordingly.

To track the visitors’ viewing behaviour, we employed a computer application which we programmed for the sake of this observation study as an Android app on Sony tablet personal computers. The application allowed the fast and intuitive entering of data—viewing distance was assessed in steps of 0.5 m corresponding to the tile structure on the floor of the exhibition hall. Times were recorded by automatic timers which were triggered by entering a viewing distance from a specific painting on the screen and were stopped by tapping on the corresponding picture. Further information could be recorded, for example, demographic data (gender and age) or whether the observed person was accompanied by other persons or showed some characteristics of interest (e.g., using a walking stick). Importantly, the app was easily capable of going back to specific persons in order to enter additional data for already observed visitors who had returned to a painting. This technique allowed the history of the viewing of such a painting to be compiled.

### Procedure

Two observers were located on the balcony, with Observer 2 assisting the experimenter Observer 1, who entered the data. This was done first of all to ensure objective data entry and was also used for tracing visitors who might potentially come back. Note: The six assessed paintings were situated at the end of the exhibition hall, near the exit—actually just one picture was hung closer to the exit gate. Still, the whole setting of the exhibition invited the reassessment of the paintings again, as there was no constraint on how to access the single artworks in a certain way. The observers tried to focus on single visitors to capture their entire viewing behaviour with regard to the paintings under observation. This made it necessary to test single, randomly chosen persons in depth, so the duration of the total testing was considerably long as many visitors take quite a while to wander through the whole exhibition. In fact, we tested over about five consecutive weeks, from 17 January until 22 February 2015. Had an observed visitor looked above and detected the experimenters, the trial would have been abandoned and the respective data omitted from analysis, but this case never happened. Age was estimated at a resolution of 5 years; gender was assessed by the outward appearance of the person.

## Results

**Figure 2.**
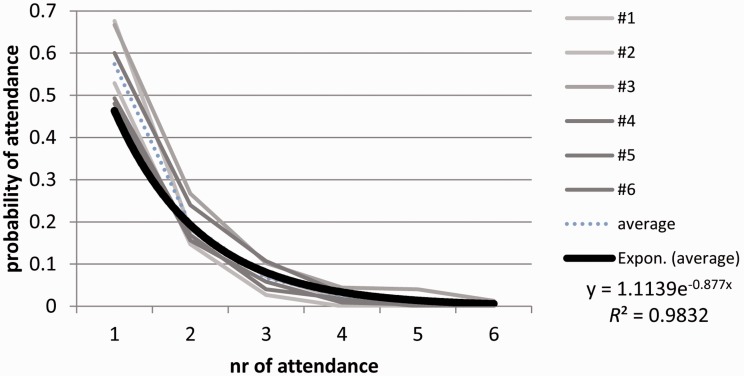
Probability of viewing (*attendance*) from the first up to the sixth viewing for the corresponding artworks no. 1 to 6. The thick black line shows an exponential fit of the average probability of attendances (the dotted blue line shows the respective average empirical data). The decrease in total number of attendances very closely followed a negative exponential function (*r*^2 ^= .983).

For data analysis, the general ideas proposed by Smith and Smith (2001) will be followed in order to provide an opportunity to compare the results across studies; this is especially important because the Smith and Smith study is often referred to as the reference study for viewing behaviour toward artworks in museums. Afterwards, additional data will be presented on viewing distance. Importantly, more than half of the visitors (55.3%) who were registered to have viewed an artwork at least once returned to it after a while—looking a little closer at the pattern of returning reveals that some visitors viewed an artwork several times; up to six times in certain cases (see Figure 2).

Therefore, the analyses will only start with a focus on the first viewing in order to be in accordance with the data report of the Smith and Smith study and will then move on with further analyses on the viewing behaviour across the several viewings: Important information on the process and nature of art experience in a museum which the original study from 2001 did not contain.

### Viewing Time of Artworks

For the following analyses, we will first focus only on the initial viewing of an artwork. Later on, this view will also be expanded upon by analysing data for visitors who came back to an artwork.

We observed quite similar viewing times to what previous research had, which is also reflected by typical experiences when art museums are attended (see detailed data in Table 2). Visitors spent on average 33.9 s (*Mdn* = 25.1 s)—Smith and Smith (2001), for instance, revealed a relatively similar mean time of 27.2 s (*Mdn* = 17.0 s). People were found to spend very different amounts of time in front of different artworks, here between 25.7 s and 41.0 s on average—note: although the exact durations differed from the Smith and Smith study, they also documented such a various viewing behaviour with a range of viewing duration from 13.2 s to 44.6 s.

**Table 2.** Mean Values for Viewing Time and Viewing Distance (Employing Only First Attendances to Artworks), Total Viewing Time (Sum of All Viewings), Mean Visual Viewing Angles Across All Viewings (*Attendances*), % of Visitors Who Attended the Artwork At All, % of Persons Attending Who Returned At Least Once to the Artwork, Times of Viewing if a Person Had Viewed the Artwork At All.

No.	Viewing time (first)	Viewing time (total)	Viewing distance (first)	Viewing distance (mean)	Viewing angles (mean) W × H	% of visitors attended	% of visitors came back	Mean number of viewings if attended at all
1	29.6 s	36.3 s	1.60 m	1.59 m	42.7° × 34.7°	52.9	45.6	1.33
2	30.0 s	42.2 s	1.57 m	1.56 m	25.8° × 36.0°	67.6	48.4	1.41
3	39.2 s	64.2 s	2.12 m	2.03 m	50.4° × 41.3°	66.7	64.7	1.70
4	41.0 s	67.2 s	2.05 m	2.10 m	51.8° × 37.7°	60.0	63.8	1.66
5	25.7 s	38.5 s	1.66 m	1.55 m	18.8° × 16.5°	49.3	54.9	1.46
6	36.1 s	47.6 s	1.49 m	1.45 m	19.4° × 26.7°	48.0	54.1	1.47
Mean	33.9 s	50.5 s	1.75 m	1.72 m	37.6° × 33.5°	57.4	55.3	1.51

*Note.* Average values in the last rows are based on arithmetic means of the participant-oriented values and thus differ from simple unweighted mean values of the six artworks.

Visitors viewed the artworks quite selectively, omitting 2.5 out of the given range of six pictures—a clear sign of selective viewing behaviour even in a special art exhibition showing a very limited number of paintings.

In contrast to Smith and Smith (2001), we did not find any substantial differences among group sizes, *F*(3, 221) = 1.16, *p* = .3271, *ns*: Category *single* visitors showed a mean viewing duration at first attendance of 35.6 s, category *pair* showed 31.4 s, *group* showed 36.5 s, and *family* showed 36.4 s. In accordance with the Smith and Smith study, we could not find any significant difference between female (*M* = 34.6 s) and male visitors (*M* = 32.7 s), *t*(214) < 1, *p* = .5601, *ns*.

When returns to the artworks are taken into account, the entire data pattern changes in many respects. First of all, taking the total viewing duration comprising all viewings of an artwork together, we get much longer viewing times than those observed by Smith and Smith (see details in Table 2): Given this total viewing time perspective, visitors spent 50.5 s on one artwork. In fact, visitors who viewed an artwork at least once showed a 51% probability of returning to it at least once more. The probability of viewing in relation to the number of viewings decreased systematically following an exponential function (see Figure 2) with a very high fit of *r*^2 ^= .9832.

**Figure 3.**
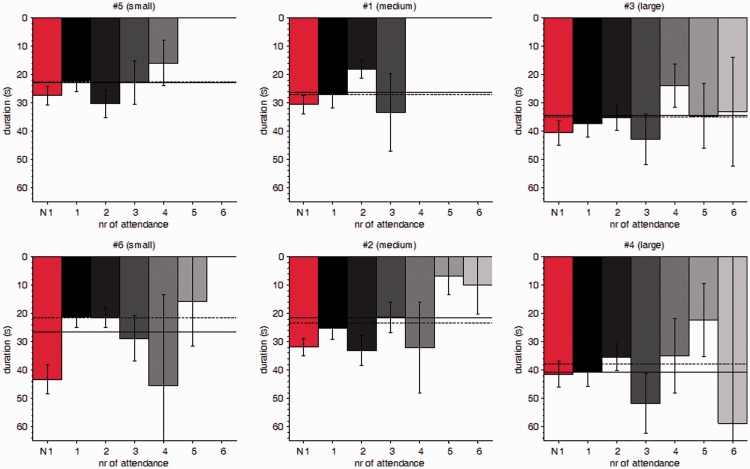
Viewing durations (in seconds) of all pictures (plots are grouped based on the raw area sizes of the pictures, i.e., small, medium, and large). The bars provide data on viewing durations for the respective viewing session (e.g., 1 = first viewing of the picture); N1 (“Non-return 1^st^,” red bars) gives all initial viewings of a picture in which people did *not* return to the respective picture. Error bars indicate ±1 standard error of the mean.

It is also worth noting that initial viewings of artworks which were later viewed at least one more time (*M* = 25.8 s) were viewed for a much shorter period than artworks which were viewed only once (*M* = 37.9 s), *t*(128) = 4.024, *p* < .0001, *d* = 0.268. It appears people who came back felt like returning quite quickly when inspecting the artwork initially; or probably, they realized while looking one of the next paintings that they had looked at the previous artwork too briefly. This effect can be observed when inspecting the graphical data in Figure 3 by comparing the red and the solid black bars which correspond to the initial viewings of a picture which people never returned to, or returned to at least once, respectively.

Furthermore, in taking total viewing times into consideration instead of just the initial viewings of artworks, we indeed obtained differences among groups, *F*(3, 221) = 3.00, *p* = .0315, η_p_^2 ^= .039, in a very comparable fashion to Smith and Smith, although the absolute times were much longer than in the original study: *single* (*M* = 46.2 s), *pair* (*M* = 53.3 s), and *group* (*M* = 73.4 s). Interestingly, our additional category *family* showed the shortest total viewing times (*M* = 40.7 s) of all of the groups, speculatively, because attending children mostly tend to distract the viewers; in fact, parents are often forced to pay more attention to the children than to the artworks they initially came for. As in the analysis on initial viewings of artworks, female (*M* = 51.4 s) and male visitors (*M* = 50.8 s) did not differ in total viewing times, *t*(214) < 1, *p* = .8588, *ns*.

### Viewing Distance From Artworks

**Figure 4.**
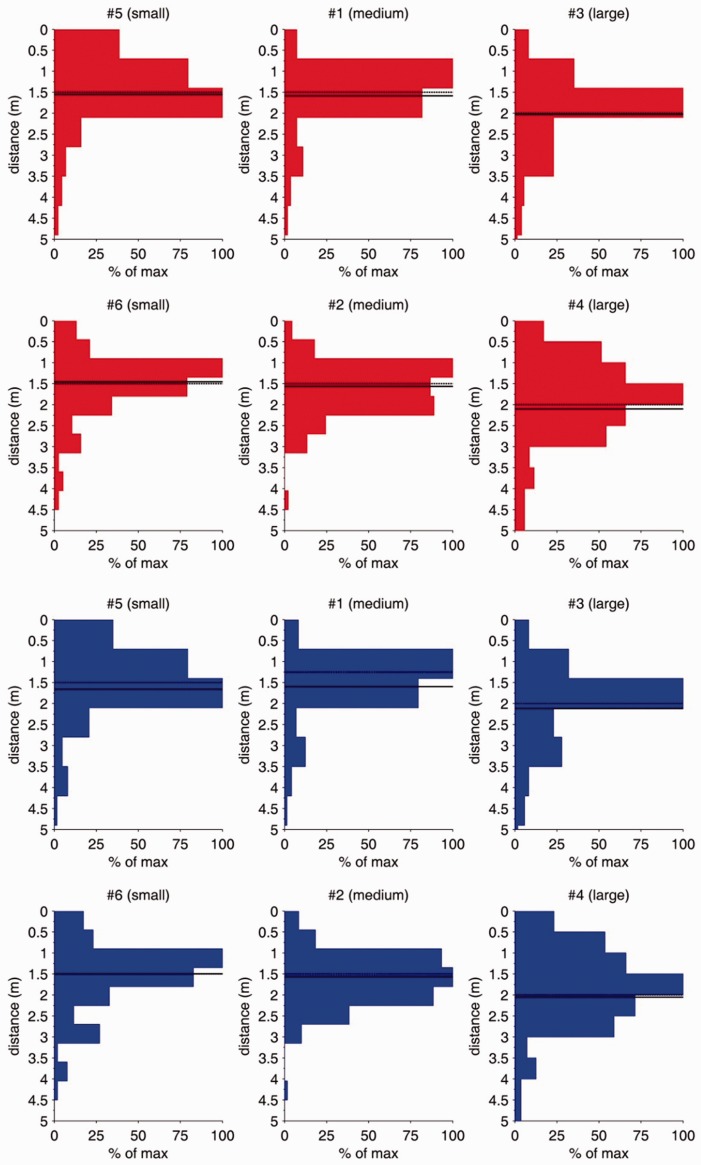
Histograms of viewing distances (in metre) for all used pictures plus mean (thicker, solid line) and median values (thinner, dotted line) for initial viewings only (red bars; top 2 rows) and for all viewings aggregated (blue bars; bottom 2 rows). The plots are grouped based on the raw area sizes of the pictures (small, medium, and large).

As documented in Table 2, viewing distance was very different to typical viewing conditions in the lab. Whereas typical distances in the lab are mostly constrained by the testing scenario with a person sitting in front of a computer screen or a pile of postcards with a very short viewing distance of about 50 to 60 cm—and in many eye-tracking scenarios even closer than this due to optimization of the input signals—we observed a mean viewing distance of 1.75 m at initial viewings of the pictures, ranging from 1.49 to 2.12 m. Locher et al. (2001) also observed viewing distances from artworks in a museum being mostly larger than in typical lab settings (with a range of between 60 and 120 cm). Interestingly, the lower limit of viewing distance was observed when visitors viewed a quite small-sized painting (i.e., “Portrait of a Carthusian” by Early Dutch painter Petrus Christus in 1446, sized W × H = 21.6 × 29.2 cm [indicated as W = 20.3 cm in the original publication by Locher et al. (2001)]). This is very similar in size to an A4 sheet of paper, yielding visual angles of 20.4° × 27.3°—which is quite compatible with the viewing conditions of typical computer testing scenarios in the lab. So at least with such smaller sized pictures, the viewing conditions in terms of the visual angle seem to be quite compatible between a lab and museum context. This could even be the reason why many people feel immersed in a lab context (known as the facsimile accommodation effect, see Locher, Smith, & Smith, 1999); at least, when such smaller sized pictures are depicted on the display. Locher et al. (2001) also revealed that the largest painting in the tested museum setting (“Aristotle with a Bust of Homer” by Rembrandt, sized W × H = 136.5 × 143.5 cm) yielded the biggest viewing distance of 1.2 m, resulting in visual angles of W × H = 59.0° × 61.4°. Although the latter visual angles are much larger than the ones revealed by the present study, they probably point in the same direction of conclusion: If people are allowed to adjust their viewing distance on their own, they tend to use larger distances for bigger pictures. For an illustration, see the histograms of the viewing distances of the present study in Figure 4 grouped by raw categories of the area sizes of paintings (small, medium, and large).

**Figure 5.**
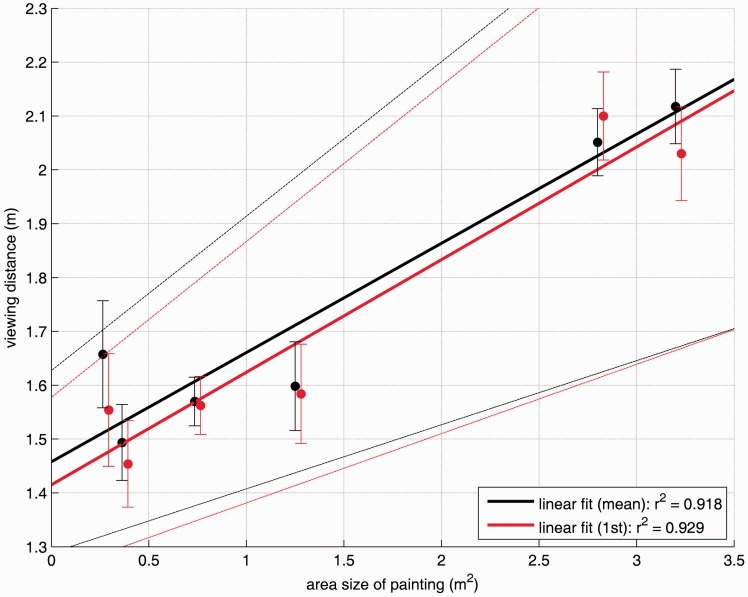
Relationship between area size of paintings (in square metre) and the self-chosen viewing distance (in m). Black solid circles indicate empirical data for the mean distance of all viewings; red solid circles only show the empirical data for the initial viewings of a picture. Regressions lines are based on *N-*weighted simple linear regressions, dotted lines indicate 95% confidence interval limits.

To test this more systematically, a simple linear regression with weights based on the respective *N* was conducted with the area size of the painting (in square metre) as independent and the viewing distance as dependent variable. As illustrated in Figure 5, a clear linear relationship was obtained: When using the viewing distances aggregated over all attendances with *r*^2 ^= .918 (distance = 1.458 m + 0.203 × picture size in m^2^) and a similar close fit (*r*^2 ^= .929) when using the viewing distances of first attendances only (distance = 1.415 m + 0.209 × picture size in m^2^), these models indicate that the visitors confronted with paintings by Gerhard Richter in this specific art exhibition used a minimum distance of about 1.4 m and their distance increased by about 20 cm per square meter of a painting. Alternative models, for instance, using different dependent variables such as the visual angles for the *x* axis or the visual angles for the *y* axis, or using different independent variables such as the width or the height of the paintings, did not outperform this very close fit.

Finally, we did not find any substantial differences among group sizes and between gender, neither for aggregated data across several viewings nor for just the data on the initial viewings of the pictures.

## General Discussion

The main aim of the present study was to add insights into ecologically valid behaviour of art perceivers in a museum context. Based on the seminal article by Smith and Smith (2001) of visitors’ viewing times for paintings in the permanent collection of the MET in New York, an extended approach comprising additional measurements was employed during a temporary Gerhard Richter exhibition. The main expansion was made by complementing the observation data by analysing the viewing distance and visitor behaviour across different viewings of an artwork.

The viewing times revealed in this temporary exhibition, ranging on average between 25.7 and 41.0 s per picture, were clearly longer than those of the original study by Smith and Smith (*M* = 27.2 s); especially when the multiple viewings of an artwork were taken into account (Range: 36.3–67.2 s). One very good reason for these much longer viewing times in our study might be the specifics of the art exhibition in which the visitors were observed: (a) the testing context of our study was a very popular temporary exhibition with very well-renowned paintings by one specific artist who is among the top contemporary painters in the world, so people were viewing these specific paintings with great enthusiasm (this was indeed the feeling I had when attending the exhibition and having also talked with some visitors afterwards—note: my personal attendance at the exhibition was not part of the testing period for the observation study); it is quite probable that people who attended the present exhibition had better and more focused background knowledge of the specific artist, here Gerhard Richter, and greater possibilities to benefit from longer viewing times (see Leder et al., 2006); (b) the temporary exhibition only contained 28 works in total compared with the volume and extreme variety of over a million artefacts exhibited at the MET where Smith and Smith (2001) made their observations. Jeffrey and Lisa Smith, at that time both affiliated to the MET—and thus being real experts on this specific museum—mentioned that typical visitors spend only about 2.5 to 3 h in the whole museum; furthermore, in what is now known as the *Museum effect*, most visitors spend their time in the whole museum, without just viewing a short list of individual artworks, resulting in viewing times of less than 1 min per artwork (Smith, 2014). This inevitably results in a rather shallow inspection of many artworks. In the present study, visitors viewed a given artwork for approximately 50 s but also had only 28 paintings in the whole exhibit to elaborate on. This mere fact might be one of the essential success factors of temporary exhibitions, which show a very limited number of artworks: People can really focus on one topic and elaborate this. With such a limited set of works they might be better able to gain Aesthetic Aha-insights (Muth & Carbon, 2013), pleasure (Biederman & Vessel, 2006), and sustainable memories (Medved, Cupchik, & Oatley, 2004) though deeper processing, even of innovative, unknown, atypical, or challenging artworks (Belke, Leder, & Carbon, 2015). In this respect, we should also argue against economic wisdom, but that visitors should be invited and allowed to spend a lot more time with highly selected artworks. This would enable them to really take the thrilling journey of dynamic aesthetic experiences (Muth, Hesslinger, & Carbon, 2015)—such “truly aesthetic experiences” are characterized by oscillating between initial rejection, allegedly full understanding, interest–disinterest and final insights at least in part, which is typical for many artworks, especially contemporary ones that contain semantic instability (*SeIns*, Muth & Carbon, 2016). In the end, *SeIns* and the ongoing dynamic course of processing makes art so unique and so sustainably interesting, because even having found one answer to a question, an artwork keeps asking multiple new questions as a reaction to the alleged one answer.

In our observation study, we also registered reviewing of artworks which underline the dynamic character of art experience; actually, visitors came back to an artwork in more than 50% of cases—these data are very compatible with a recent article that analysed the wandering behaviour of museum visitors over time as being quite complex, fuzzy, and characterized by multiple viewings of artworks (Tröndle, Wintzerith, Wäspe, & Tschacher, 2012). Interestingly, we observed a systematic difference in viewing times if an artwork was only viewed once and if further viewings were registered later on. Whether visitors already planned to return while initially looking at the painting we can only speculate, but it is a fact that if so, then they initially spent a substantial period of 12 s less in viewing the painting. This could be a kind of a strategic precheck or a screening mode for tagging the artworks onto a list of paintings which attracted the respective visitor in a certain way. Possible attractors could be manifold; for instance, mere personal preference for specific paintings or, even more plausible, interest in them (see Cupchik & Gebotys, 1990; Silvia, 2006), often triggered by cognitive challenge (Muth et al., 2015) due to ambiguity (Zeki, 2004) or indeterminacy (Pepperell, 2011)—all these properties refer to psychological aspects of art experience guided by situationally modulated affordances (see Trondle, 2014), but do not refer to prefixed object-based properties (Carbon, 2011). Such returns to paintings which have previously been tagged seem to provide quite interesting observation phenomena. First of all, people reviewed these paintings voluntarily, suggesting that they showed something personally significant; furthermore, the processing of such artworks might be qualitatively different as they were not only viewed longer but were also viewed from potentially different distances and perspectives and with the chance of having further *elaborated* (Carbon & Leder, 2005) or *incubated* (Medved et al., 2004) some insights in between viewings.

Regarding the different viewing distances at which visitors choose to inspect the paintings, we again observed that conditions were very different to the typical ones employed in lab research. On average, the visitors in the present study distanced themselves from a painting *M* = 1.72 m across all viewings, which was not substantially different from the distance they used when only initial viewings were analysed (*M* = 1.75 m). First of all, the essential difference between a museum and a lab context is mainly that a museum offers enough space for visitors to choose their personal distance from an artwork. On what basis visitors choose *their* distance remains unclear, but it is seemingly done by intuition without any deeper rationale behind it. This intuition seems to have a basis in the extension of the artwork, here the canvas size: The larger the artwork the more viewing space is chosen. It is important to note that the revealed linear relationship with a very close fit between data and model was achieved at a very specific art exhibition devoted to one painter *only*; it should, however, also be noted that Gerhard Richter’s oeuvre is definitely a very wide, rich, and diverse one, and even the selection of six paintings here showed some obvious diversity in style and age. Although an exhibition displaying much more heterogeneous styles of various artists from different cultures would probably also have caused adapted parameters for the model equation, the uncovered psychophysical model—which I would like to term “art viewing distance accommodation”—needs further investigation as it potentially points to an implicit aesthetic viewing behaviour of trying to optimize the immersion and perceptual understanding of a painting.

One great difference between a museum context and a lab setting is the typical presence of many people in the same hall, the *sociality* factor (Tröndle et al., 2012)—many people view artworks with their partner, in groups or with their family, which was considered here as factor *group*. We indeed detected an effect of group which was very compatible with the obtained effect of the Smith and Smith study: Pairs of visitors took longer viewing times, often because they debated on the painting, but more than two persons attending a painting together even outperformed *pairs*. The additional categorization of *family* showed the shortest viewing times—probably due to ongoing caretaking issues, especially for parents with small children; every museum visit is a challenge of its own, characterized by a great deal of distraction and the fulfilling of secondary tasks. Similar to Smith and Smith, we were not able to find any gender effect. This finding is also reflected by a very recent forthcoming study by Smith, Smith, and Tinio (in press) which was been able to replicate most of the findings of the original Smith and Smith study in a different museum (The Art Institute of Chicago). Quite worth noting is the fact that in this updated study, many visitors spent a significant amount of time and effort posing in terms of making selfies in front of the artworks. The authors called this *arties* to give reference to this specific case of self-posing. In the Gerhard Richter exhibition utilized in the present study, photographing was strictly forbidden and, given the very transparent and easily accessible character of the whole exhibition hall, people apparently complied with this rule—and so arties, as a consequence, were not at all a factor in the present study.

## Conclusion

Once again, the present study made clear that viewing artworks in a museum context is very different to a typical lab setting: First of all, visitors of art museums invest money, time, and intellectual effort beforehand to get to the exhibition hall, they show more skills and motivation to deeply process artworks (Winston & Cupchik, 1992), and, screening the demographics of typical visitors, they are mostly older and possess more knowledge of art and so also show different heuristics in assessing the quality of art (Haertel & Carbon, 2014; Winston & Cupchik, 1992); second, the whole social setting is very different with people walking around in a relatively silent and focused—but still communicative and interactive—way (Salar, 2009). In a very real sense, the museum context can provide a situation of great *sociality* (Tröndle et al., 2012); third, the viewing distances from paintings is very different, typically larger (Locher et al., 2001), and is, not to forget, self-chosen; fourth, the viewing duration is also self-chosen and fundamentally (much) longer than in typical lab settings (Smith & Smith, 2001) providing more possibilities and ways of elaborating on the artworks; fifth, the size and quality of paintings are mostly very different, with artworks in museums being displayed in their original size (Locher & Dolese, 2004; Locher et al., 2001) and where a more holistic view of the artwork is enabled (Carbon, 2016) which has a fundamental impact on the further processing and on the viewing behaviour and elaboration of the artworks (Locher et al., 2001); and sixth and probably not least, artworks in museums refer to (mostly or at least supposedly) originals, whereas participants in a lab inspect standardized depictions of artworks, so both settings differ in influential effect caused by authenticity (Wolz & Carbon, 2014).

In conclusion, if we are really interested in deeper investigations into how art experience differs from other types of experience, we have to analyse the psychological processes with reference to original artworks in the context of art exhibitions, galleries, or private collections. Only if we do, this will we be able to reveal intuitive and spontaneous behaviour face-to-face to artworks—such interesting and psychologically insightful behaviour which we would otherwise suppress in an overly systematized and constrained lab context.
